# An Update on the Role of Adipose Tissues in Psoriasis

**DOI:** 10.3389/fimmu.2019.01507

**Published:** 2019-06-28

**Authors:** Yisheng Wong, Satoshi Nakamizo, Kahbing J. Tan, Kenji Kabashima

**Affiliations:** ^1^National Skin Centre, Singapore, Singapore; ^2^Singapore Immunology Network, Skin Research Institute of Singapore, A^*^STAR, Singapore, Singapore; ^3^Department Dermatology, Kyoto University School of Medicine, Kyoto, Japan

**Keywords:** obesity, psoriasis, adipose tissue—obesity, adipokine cytokines, adipocyte, macrophage—cell, leptin

## Abstract

Psoriasis is a common chronic inflammatory skin disease that is increasingly being recognized as a disease that not only affects the skin but also has multi-systemic implications. The pathophysiological link between psoriasis and obesity is becoming increasingly elucidated by recent studies. The cross-talk between adipocytes and the immune system via various mediators such as adipokines could explain how obesity contributes to psoriasis. The effects of obesity on adipocytes include upregulation of pro-inflammatory adipokines such as leptin and resistin, downregulation of anti-inflammatory adipokine, and also the stimulation of pro-inflammatory cytokine production by macrophages. This article provides an update on the role of adipose tissues in psoriasis.

## Introduction

Psoriasis is a common chronic inflammatory skin disease with an estimated worldwide prevalence of 0.5–11.4% in adults, and 0–1.4% in children (1). It is a hyperproliferative skin disorder with a complex immune-mediated etiology involving an interplay amongst T lymphocytes, dendritic cells, and keratinocytes via various cytokines (2–4). Scientific advancements over the past decade have empowered us with a greater understanding of the genetics, pathophysiology, co-morbidities, and treatment of psoriasis. Psoriasis is increasingly being recognized as a disease that not only affects the skin but also has multi-systemic implications. One of the comorbidities associated with psoriasis that has been rigorously studied in recent years is obesity (5). Typically defined as a body mass index (BMI) ≥30 kg/m^2^, obesity has been described as an escalating global epidemic and a serious public health concern, especially in developed nations (6). Obesity has been well-known to cause significant excess in mortality and morbidity, being associated with a myriad of obesity-related complications. Obesity increases the risks of type 2 diabetes and cardiovascular diseases. Interestingly, obesity has also been found to be an independent risk factor for the development of psoriasis (7). The pathophysiological link between psoriasis and obesity is becoming increasingly elucidated. There have been mounting evidence to show that both psoriasis and obesity represent a pro-inflammatory state, and that immunological mechanisms in both conditions have significant overlap. Contrary to prior belief that the adipose tissue plays a role only in energy storage, it is in fact a large endocrine and secretory organ that produces a multitude of pro-inflammatory cytokines and adipokines, resulting in various downstream effects (8). Adipocytes are also known to regulate inflammation even locally in the skin (9). In this mini review article, we aim to shed some light on the role of adipose tissues in psoriasis.

## Epidemiology on the Relationship Between Obesity and Psoriasis

The association between obesity and psoriasis was consolidated by a systematic review with meta-analysis of observational studies between 1980 and 2012 (5). A total of 16 observational studies with 201,831 psoriasis patients were included in this meta-analysis. Compared with the general population, psoriasis patients have higher prevalence and incidence of obesity. The pooled odds ratio (OR) for obesity among patients with psoriasis was 1.66 (95% confidence interval (CI) 1.46–1.89) compared with those without psoriasis. Another more recent systematic review confirmed that different adiposity measures such as BMI, waist circumference, waist-to-hip ratio, and weight gain positively correlated with increased risk of psoriasis (10). The summary of relative risk (RR) for a 5-unit increment in BMI was 1.19 (95% CI 1.10–1.28). The summary of RR was 1.24 (95% CI 1.17–1.31) per 10 cm increase in waist circumference, 1.37 (95% CI 1.23–1.53) per 0.1 unit increase in waist-to-hip ratio, and 1.11 (95% CI 1.07–1.16) per 5 kg of weight gain.

Studies have also shown that there is a strong correlation between severity of psoriasis and obesity (11). Patients suffering from severe psoriasis were found to have greater odds of obesity than those with mild psoriasis. A population-based study conducted in the United Kingdom showed that there was a “dose-dependent” relationship between disease severity and obesity. Among the study population, those with mild, moderate, and severe psoriasis (based on body surface area involved) had the prevalence of obesity compared with controls increased by 14, 34, and 66%, respectively (12).

Obese children are also at higher risk of developing psoriasis. In an international cross-sectional study of 409 children with psoriasis, children with psoriasis were significantly more likely to be obese than controls (OR 4.29, 95% CI 1.96–9.39) (13). In a retrospective cohort study over a 10-year period, nearly 30,000 children with psoriasis were compared with an age-, sex-, and race-matched comparator cohort without psoriasis (14). In this study, it was found that children with psoriasis had higher rates of not just obesity, but also other components of metabolic syndrome such as hyperlipidemia, hypertriglyceridemia, hypertension, and diabetes compared to children who did not have psoriasis.

Initially, the higher prevalence of obesity in psoriasis patients was thought to be solely due to the negative psychosocial aspects of having psoriasis. Psoriasis patients compared to patients without psoriasis appear to have higher rates of unhealthy behavior such as overeating, sedentary lifestyle and smoking (15). However, over the years, it has been increasingly recognized that weight gain and increased adiposity may increase the risk of developing psoriasis. In the Nurses' Health Study II, the multivariate relative risk for developing psoriasis in obese women with a BMI ≥30 kg/m^2^ was 1.73 (95% CI, 1.24–2.41) compared to only 0.76 (95% CI, 0.65–0.90) for women with a BMI <21 kg/m^2^ (7). To support the notion that obesity plays a role in development of psoriasis, a retrospective study on the pediatric population showed that being overweight or obese preceded psoriasis by at least 2 years in 93% of children with psoriasis (16).

Studies have also shown that weight loss in patients suffering from psoriasis resulted in improved severity of disease. Case reports of patients with severe psoriasis who achieved remission after successful weight loss by post-bariatric surgery alluded to the fact that weight loss could potentially be an adjunctive treatment for psoriasis (17, 18). This was supported by a later retrospective study of 34 psoriasis patients who underwent weight loss surgery, which showed that almost two-third of them experienced improvement in the severity of psoriasis after surgery (19). Moreover, weight loss seemed to have an impact on the response to treatment with systemic therapies. A randomized controlled trial of 61 patients with moderate-to-severe psoriasis who are also obese showed that there was a better treatment response to cyclosporine when it was combined with a low-calorie diet when compared with cyclosporine alone (20). It has also been shown in a large multi-center longitudinal study that in psoriasis patients on biologic therapies, higher weight was associated with reduced odds of achieving ≥90% improvement in Psoriasis Area and Severity Index (PASI 90) at 6 months post-therapy (21).

## Adipocytes in the Skin and Their Role in Immune Responses

**Figure 1:**
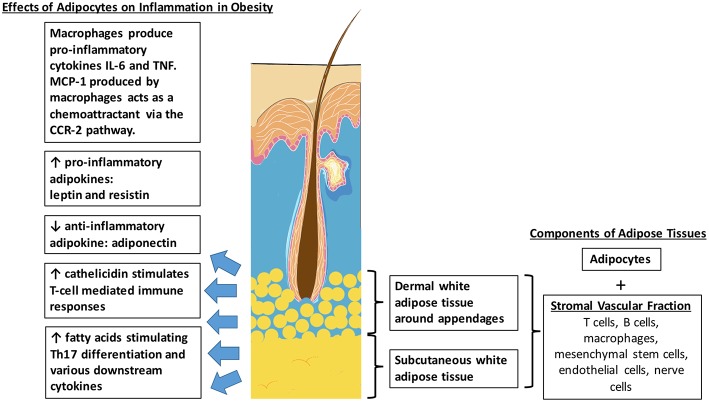
Distribution of intradermal and subcutaneous white adipose tissue. Effects of adipocytes in obesity which potentially contributes to the pathophysiological link between obesity and psoriasis.

In humans, the subcutaneous tissue forms an uninterrupted layer throughout the body with exceptions of the hands and feet, accounting for a significant 8–18% of body weight in males and for 14–28% in females, and even more in obese adults (22). The largest adipose depots are abdominal white adipose tissue. However, studies have suggested that adipose tissues also exist within the skin dermis (Figure 1) (23). In humans, it was found that there are two histologically, anatomically and metabolically distinct layers of adipose tissues beneath the reticular dermis. The nomenclature of this layer of adipose tissues between the reticular dermis and deep layers of subcutaneous tissues still remains contentious. Some groups describe it as the “superficial subcutaneous adipose tissue” whilst others are calling it “dermal white adipose tissue” (24). No matter what the nomenclature is, these adipocytes mostly reside around skin appendages, especially around hair follicles (25). Apart from mature adipocytes, adipose tissues also consist of a variety of other cell types, collectively termed the stromal vascular fraction (SVF). These include mesenchymal stem cells, vascular endothelial cells, nerve cells, macrophages, T-cells, and B-cells.

Obesity is a state of increased adiposity, resulting in changes in cell composition in adipose tissues. It is associated with increased numbers of macrophages in the SVF of both visceral and subcutaneous adipose tissues. This is supported by mouse studies which showed that that macrophages accounted for approximately 40% of the SVF in obese rodents, compared to only 10% in lean littermates. Macrophage-related genes are also found to be upregulated in these obese animals (26). Recruitment of macrophages into adipose tissues is an early event in obesity-induced adipose inflammation. The monocyte chemoattractant protein-1 (MCP-1), one of the main chemoattractants for macrophages via the C-C chemokine receptor 2 (CCR2) pathway, is secreted primarily by macrophages, vascular endothelial cells and also adipocytes (27). Adipose tissue macrophages express CCR2 and recruit additional monocytes and macrophages, promoting a feed-forward process. Activated macrophages then produce inflammatory cytokines such as IL (interleukin)-6 and TNF (tumor necrosis factor) which are known to exacerbate the symptoms of psoriasis as well (28).

Intradermal adipocytes also release high levels of antimicrobial peptides such as cathelicidin during early adipogenesis (29). Cathelicidin is found to be increased in lesional skin of psoriasis and plays an active role in inflammation. They form complexes with human self-DNA and in turn activate dermal plasmacytoid dendritic cells via toll-like receptor 9 (TLR9) to produce interferon-α (IFN-α) to stimulate T-cell-mediated immune responses (30). Serum cathelicidin protein levels were found to be significantly increased in obese, non-diabetic and pre-diabetic patients, compared with non-obese and non-diabetic patients (31). Therefore, the increased production of cathelicidin by intradermal adipocytes in obese patients could also contribute to the pathophysiology of psoriasis.

Another pathway in which adipocytes mediate immune responses is via lymphocytes. It has been shown that white adipose tissue acts as a reservoir for memory T-cells, a major component of the adaptive immune system (32). Lymphoid clusters were also found to be present in both human and mouse mesentery. Cells in these clusters proliferate in response to IL-2 and produce large amounts of cytokines such as IL-5, IL-6 and IL-13, modulating inflammation (33). These lymphocytes found in adipocytes can thus represent another factor that triggers the inflammatory response in psoriasis.

## Adipokines as a Pathophysiological Link Between Psoriasis and Obesity

**Figure 2:**
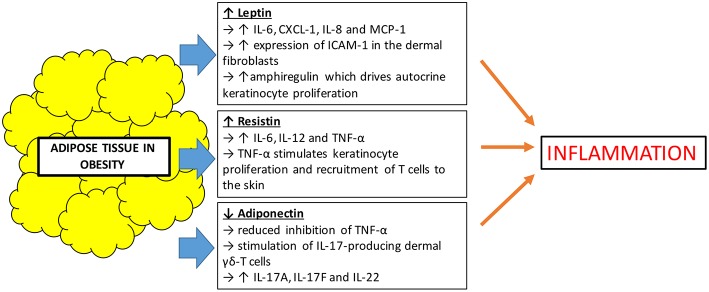
Effects of adipokines in obesity leading to inflammation.

Besides serving the role as an effective lipid storage organ and a structure for insulation and mechanical support, it has been recognized that adipose tissues also act as an active secretory endocrine organ. Adipokines are a collective term used to describe bioactive proteins produced by adipose tissues which regulate various metabolic functions including lipid and glucose metabolism, inflammation, vascular homeostasis, and coagulation (34). Obesity leads to an overproduction of pro-inflammatory adipokines and the simultaneous reduction in the production of adipokines with anti-inflammatory properties. This creates a dysregulation in the function of adipokines, which influences local and systemic inflammation (35). In obesity, there is an expansion of white adipose tissue which is the primary site that produces adipokines such as leptin, resistin, and adiponectin. These represent the three most studied adipokines. Leptin and resistin are pro-inflammatory adipokines, whilst adiponectin has anti-inflammatory effects via inhibition of TNF-α (36) (Figure 2).

First discovered in 1994, leptin is the first adipokine identified and serves as a satiety factor, regulating food intake and energy expenditure, thus coordinating changes in energy balance and whole body nutritional status (37). Leptin is a product of obese gene and is produced mainly by adipocytes. It acts on leptin receptors and exerts a multitude of effects. Apart from its primary role in regulating satiety, leptin plays a part in bone metabolism and immune functions (38). Large adipocytes produce more leptin than small ones, and it is well-established that serum leptin concentrations are strongly correlated with overall body fat content (39). Leptin is not only increased in obesity, but also found to be increased in patients with psoriasis, positively correlating with increasing severity of disease (40, 41). Understanding leptin's immunomodulatory function may help to explain its link to psoriasis. Leptin induces the production of IL-6, chemokine (C-X-C motif) ligand-1 (CXCL-1), IL-8, and monocyte chemoattractant protein-1 (MCP-1) and also the increased expression of intercellular adhesion molecule-1 (ICAM-1) in the dermal fibroblasts (42). IL-6, CXCL-1, and IL-8 may contribute to the hyperproliferative state of the epidermis in psoriatic skin. It has also been postulated that leptin induces psoriasis skin to produce amphiregulin, which is known to drive autocrine keratinocyte proliferation in culture (43). Although there has been contradicting studies showing leptin levels that are both increased or decreased in patients with psoriasis, a recent meta-analysis of 26 studies concluded that patients with psoriasis had higher leptin concentrations compared to the control population (44).

Resistin is an adipose-derived cysteine rich adipokine which not only helps in the regulation of glucose metabolism, but also has a role in inflammation. Like leptin, resistin is positively correlated with obesity. Resistin influences inflammation by inducing the release of pro-inflammatory cytokines (45). Studies have shown that resistin was also found to be present in higher concentrations in patients with psoriasis compared to control population. At the same time, there were also suggestions that increased levels of resistin were associated with increased severity of psoriasis (46). However, published studies on the association of resistin and psoriasis also had contradictory results. A recent meta-analysis attempted to demystify these inconsistencies and showed that indeed, higher serum concentrations of resistin level positively correlated with psoriasis disease progression (47). Resistin plays a pro-inflammatory role by stimulating the production of cytokines such as IL-6, IL-12, and TNF-α through the nuclear factor-kB signal pathway in human macrophages and peripheral mononuclear cells (48, 49). TNF-α is known to be able to stimulate keratinocyte proliferation and also recruitment of T-cells to the skin, therefore propagating the inflammatory pathway of psoriasis.

On the other hand, adiponectin is an anti-inflammatory adipokine which is secreted exclusively by adipose tissues. It enhances lipid metabolism by increasing lipid clearance from plasma and also helps in improving glycemic control (50). Unlike leptin and resistin, adiponectin is decreased in obese individuals compared to lean individuals (51). It is also found to be decreased in psoriasis patients when compared with control populations (44, 52). It has been shown that adiponectin has anti-inflammatory properties in keratinocytes *in vitro*, resulting from the inhibition of TNF-α. It is shown in a mouse study that lack of adiponectin exacerbates psoriasis-like skin inflammation with excessive infiltration of IL-17-producing dermal γδ-T cells (53). The inflamed skin of these adiponectin-deficient mice also expressed upregulation of Th17-related cytokines, IL-17A, IL-17F, and IL-22. Since IL-17 also plays a crucial role in the pathogenesis of psoriasis, it can be postulated that low levels of adiponectin, such as in obese individuals, could drive the inflammation in psoriasis.

Apart from the major adipokines discussed above, other adipokines that had been studied in psoriasis include the pro-inflammatory adipokines chemerin, lipocalin-2 and visfatin, as well as the anti-inflammatory adipokine omentin. A recent meta-analysis by Bai et al. (54) showed that serum levels of chemerin and lipocalin-2, like resistin, were also increased in psoriasis patients compared to healthy controls. However, differences in pooled serum levels of visfatin and omentin in psoriasis patients were not found to be significantly increased or decreased, respectively.

## Role of Fatty Acids on Psoriasis

Another possible explanation for the link between obesity and psoriasis would be fatty acids (55). Fatty acids found in high fat diet, such as saturated fatty acids and trans fatty acids, are predominantly derived from adipose tissues and provide an important source of energy for metabolically active tissues in the body. In recent years, studies have shown that fatty acids also have the ability to influence inflammation in the body via various mechanisms. Fatty acid metabolism is intimately connected to T-helper cell 17 (Th17) function, which plays a pivotal role in psoriasis as well (56). High fat diet induced expression of enzymes involved in fatty acid metabolism such as acetyl-CoA carboxylase 1 (ACC1), which therefore augments Th17 differentiation. Inhibition of fatty acid synthesis conversely reversed the obesity-induced increase of Th17 differentiation (57). Th17 then interacts with keratinocytes, endothelial cells and various immune cells including dendritic cells and neutrophils, driving the pathogenesis of psoriasis. Reactivation of memory Th17 cells is apparently responsible for chronic course of psoriasis (58). Various other studies support the fact that fatty acids play a role in psoriasis. Mouse psoriasis models induced by imiquimod demonstrated that the severity of disease was strongly correlated with free fatty acids concentration (59). Moreover, these obese mice, when compared to their lean counterparts, have an increased expression of IL-17A and IL-22 in the skin (60). Zhang et al. (61) reported that mice fed with high fat diet for 6 months spontaneously developed skin lesions. Inflammasome-related cytokines IL-1β and IL-18 were significantly upregulated in lesional skin compared to normal skin tissue, alluding to the fact that inflammasome signaling is somehow involved in the development of these skin lesions. Interestingly, the same study also showed that mice deficient in fatty acid binding protein, a transporter of fatty acids, were resistant to high fat diet induced skin lesions. A recent study also showed that resolvin E1 (RvE1), an omega-3 poly-unsaturated fatty acid (PUFA)-derived metabolite, potently suppressed the inflammatory cell infiltration and epidermal hyperplasia in an imiquimod-induced mouse psoriasis model. The study showed that RvE1 inhibited IL-23 production by dendritic cells and exerted inhibitory effects on migration of cutaneous dendritic cells and γδ T cells (62). Therefore, omega-3 PUFA derivatives may potentially be developed as a novel therapeutic agent for psoriasis.

## Conclusion

The relationship between psoriasis and obesity is a complex one and recent studies described above have enabled us to better understand their pathophysiological link. The cross-talk between adipocytes and the immune system via various mediators such as adipokines could explain how obesity contributes to psoriasis.

## Author Contributions

YW wrote the first draft of the manuscript. All authors contributed to manuscript revision, read, and approved the submitted version.

### Conflict of Interest Statement

The authors declare that the research was conducted in the absence of any commercial or financial relationships that could be construed as a potential conflict of interest.
